# Influence of Huangqin Decoction on the immune function and fecal microbiome of chicks after experimental infection with *Escherichia coli* O78

**DOI:** 10.1038/s41598-022-20709-3

**Published:** 2022-10-05

**Authors:** Junyan Wang, Rui Li, Minai Zhang, Chensheng Gu, Haili Wang, Jianjian Feng, Linjie Bao, Yihe Wu, Shuming Chen, Xichun Zhang

**Affiliations:** 1 College of Veterinary Medicine, Shanxi Agricultural University, Taigu, People’s Republic of China; 2 College of Pharmaceutical & Food Engineering, Shanxi University of Chinese Medicine, Yuci, People’s Republic of China; 3 Technical Center of Taiyuan Customs, Taiyuan, Shanxi People’s Republic of China

**Keywords:** Drug regulation, Medicinal chemistry, Pharmacology, Drug discovery

## Abstract

Huangqin Decoction (HQD), a traditional Chinese medicine formula from the *Shang Han Lun* written by *Zhang Zhongjing*, has been used in China for nearly two thousand years. According to the traditional Chinese medicine and previous literature, HQD has the effect of clearing heat, removing toxins, relieving diarrhea and pain. Therefore, HQD was used to prevent or cure many diseases, such as inflammation, diarrhea, malaria, and other acute or chronic gastrointestinal diseases. The effect of HQD, one-herb-absent HQD treatments and enrofloxacin (ENR) on the average daily gain (ADG), mortality rates, visceral index and toll-like receptors (TLRs), inflammatory factors and intestinal microflora in *E. coli* O78*-*inoculated chicks were investigated. HQD supplementation increased ADG and reduced the mortality rates caused by *E. coli* challenge, decreased the heart, liver, bursa of Fabricius (BF) and spleen index. HQD supplementation decreased the serum lysozyme (LZM), IL-1β, TNF-α, IL-10, IL-6 level, down-regulated the mRNA expression of TLR4, -5 and -15 in the spleen by *E. coli* challenged chicks, and up-regulated the mRNA expression of TLR4, -5 and -15 in BF. At the phylum level, HQD supplementation reversed the increase of Operational Taxonomic Unit (OTUs), decreased the relative abundance of harmful bacteria *Proteobacteria*, increased the relative abundance of probiotic bacteria *Bacteroidetes* and *Firmicutes*. At the genus level, HQD decreased the relative abundance of harmful bacteria *Escherichia-Shigella* and *Pseudomonas*. It means that HQD treatment reversed the change of the gut microbiota structure. Compared with HQD, HQD-DZ and HQD-HQ increased the mortality rates. HQD-HQ decreased the ADG and liver index. HQD-GC decreased the spleen index. All herb-absent increased the serum IL-6, but only the HQD-HQ and HQD-SY increased the serum TNF-α. All herb-absent did not activate the TLRs signaling pathways in spleen and BF of chicks. The harmful bacteria *Escherichia-Shigella* were increased in HQD-HQ and HQD-DZ treatments. HQD-DZ treatment also increased the level of *Proteobacteria.* The results showed that dietary supplementation with HQD, by down-regulating the mRNA expression of TLR4, -5 and -15 in the spleen, further decreasing the serum LZM and IL-1β, TNF-α, IL-10, IL-6 level, improves the immune function and reverses the change of fecal microbiome in chicks challenged with *E. coli*. In herb-absent supplementation, the results showed that SY and DZ play a key role in reducing the levels of inflammatory factors and keeping fecal microbiome balance respectively. More importantly, HQ is indispensable in HQD, not only play a key role in reducing the level of inflammatory factors, but also in keeping the balance of fecal microflora.

## Introduction

Avian pathogenic *E. coli* (APEC), gram-negative bacteria, are causative agents associated with septicemia. Colibacillosis refers to any localized or systemic infection caused by APEC of specific serotypes or by opportunistically pathogenic *E. coli*, causing one of the crucial bacterial diseases of poultry^1^. Generally, APEC colonizes and invades epithelial cells, are mostly associated with extraintestinal disease, principally respiratory or systemic infections and sepsis^2^. *E. coli* infection in chicks led to severe diarrhea, decreased feed intake, and reduced growth performance^3^. Chicks, in which the protective immune system is not fully developed, are more vulnerable. A limited number of serotypes, principally *E. coli* O1, -O2, -O78, -O8, and -O35 are commonly implicated in avian colibacillosis^4^. Although various antibiotics are typically used to prevent and control colibacillosis, cumulative reports have demonstrated that drug resistance of *E. coli* O78 has increased owing to the spread of resistance genes such as extended spectrum beta-lactamases (ESBL) and/or plasmid-mediated Amp-C beta-lactamases (Amp-C)^5^. Therefore, potential antibiotic alternatives to reduce antimicrobial drug usage in poultry production are urgently needed.

HQD, a traditional Chinese medicine formula from the *Shang Han Lun* written by *Zhang Zhongjing*, consists of four components: Chinese skullcap (*Scutellaria baicalensis*), white peony root (*Paeoniae radix alba*), jujube (*Ziziphus jujuba*), and licorice (*Glycyrrhiza glabra*). Clinical studies have shown that HQD is safe and effective for treatment of complex gastrointestinal symptoms, like ulcerative colitis and associated cancer^6^. The researchers suggest this is because HQD could regulate the structure of intestinal flora and inhibit the intestinal inflammatory signaling pathway^7^. The main effectors of HQD are baicalin, paeoniflorin, polysaccharides and flavonoids, which can regulate the number of mast cells and down-regulate the expression of inflammatory factors through the activation of TLR4/MyD88/NF-κB, IL-6/JAK/STAT3 and STAT3/NF-κB/IL-6 signaling pathways^8^, thus play an important role in the immune regulation and have an anti-inflammatory effect^9^. HQD has no inhibitory effect on *E. coli* in vitro, but the antibacterial effect was enhanced after intestinal flora metabolism^10^, indicating that the bacteriostatic effect of HQD is mostly dependent on regulation of the metabolites and balance of the intestinal bacteria, rather than direct effect on the pathogenic bacteria. Therefore, this study hypothesized that the HQD is effective for treatment of chicks colibacillosis by regulating the immune function and intestinal microflora structure.

To investigate the molecular mechanisms of HQD treatment in vivo, the chicks were experimentally inoculated with *E. coli*. The expression levels of LZM, inflammatory cytokines in serum, and TLRs in spleen and BF were measured. Furthermore, the gut microbiota was profiled using Illumina MiSeq 2500 sequencing of the bacterial 16S rDNA gene V3-V4 region.

## Results

### The mortality rates and average daily gain

**Figure 1:**
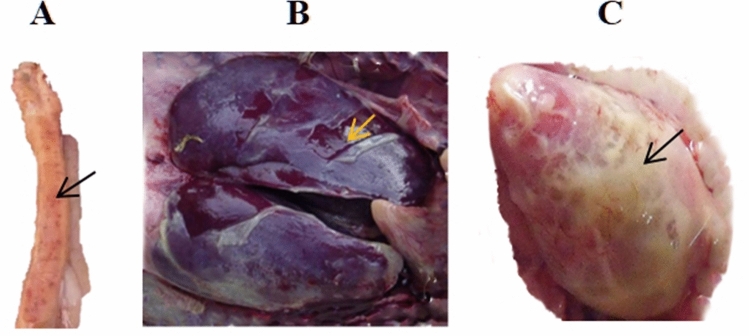
Colibacillosis chicks show severe intestinal bleeding site (**A**), perihepatitis (**B**) and pericarditis (**C**).

Compared with the CG, chicks challenged with *E. coli* at 27 days of age showed ruffled feathers, closed eyes, reluctance to move and defecated white or green feces with soiled vents. Post-mortem examination showed that *E. coli* infection led to severe perihepatitis, pericarditis, and bleeding spots in the small intestine (Fig. 1).

**Figure 2:**
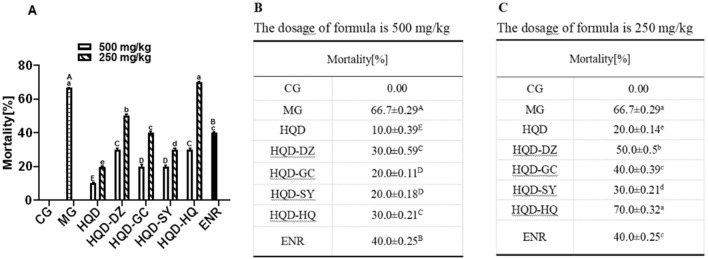
Mortality of chicks were represented in (**A**–**C**). The different letter superscript in the same column indicates that the difference is significant (*p* < 0.05). CG, Control group; MG, Model group; DZ, jujube, *Ziziphus jujuba*; HQ, Chinese skullcap, *Scutellaria baicalensis*; GC, licorice, *Glycyrrhiza glabra*; SY, white peony root, *Paeoniae radix alba*; HQD, decoction consists of DZ, HQ, GC and SY; HQD-DZ, HQD absent of DZ; HQD-GC, HQD absent of GC; HQD-SY, HQD absent of SY; HQD-HQ, HQD absent of HQ; ENR, 10 mg/kg Enrofloxacin.

In MG, the mortality rates was significantly increased (*p* < 0.05), when compared with the CG. Compared with the MG, the mortality rates was significantly decreased (*p* < 0.05) in HQD granules 250 and 500 mg/kg·BW, HQD-GC granules 500 mg/kg·BW and HQD-SY granules 500 mg/kg·BW. However, there was no significant difference in HQD-GC granules 500 mg/kg·BW and 250 mg/kg·BW, HQD-SY granules 500 mg/kg·BW and 250 mg/kg·BW. Therefore, the dosage of 250 mg/kg·BW groups were not analyzed, while the dosage of 500 mg/kg·BW groups were more worthy of further study (Fig. 2).

**Figure 3:**
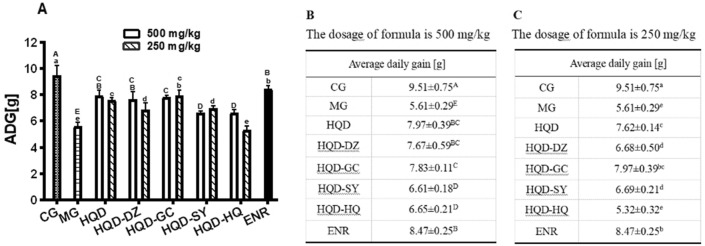
Average daily gain of chicks were represented in (**A**–**C**). The different letter superscript in the same column indicates that the difference is significant (*p* < 0.05). CG, Control group; MG, Model group; DZ, jujube, *Ziziphus jujuba*; HQ, Chinese skullcap, *Scutellaria baicalensis*; GC, licorice, *Glycyrrhiza glabra*; SY, white peony root, *Paeoniae radix alba*; HQD, decoction consists of DZ, HQ, GC and SY; HQD-DZ, HQD absent of DZ; HQD-GC, HQD absent of GC; HQD-SY, HQD absent of SY; HQD-HQ, HQD absent of HQ; ENR, 10 mg/kg Enrofloxacin.

In MG, the ADG between 1 and 32 days of age was significantly decreased (*p* < 0.05), when compared with the CG. Compared with the MG, the ADG between 1 and 32 days of age was significantly increased (*p* < 0.05) in HQD treatment. There was no significant difference between HQD-HQ, HQD-DZ, HQD-GC, HQD-SY and ENR supplementation compared with HQD (Fig. 3).

### Visceral index

Compared with CG, the heart index and the liver index were increased (*p* < 0.05) in MG, which was reversed by HQD and ENR treatments. Compared with the CG, the BF index was significantly decreased (*p* < 0.05) in MG, however, which was reversed by supplementation of HQD and ENR treatments. The chicks in MG showed no effect on spleen index, while the same index was increased by the HQD supplementation. HQD-DZ and HQD-SY have the same effect as HQD, HQD-GC resulted in decreased spleen index, while HQD-HQ resulted in increased liver index in chicks challenged with *E. coli* (Table 1).

**Table 1:** Visceral index of chicks were shown in the table.

	Spleen	Bursa of Fabricius	Heart	Liver
CG	1.89 ± 0.44^b^	5.84 ± 0.25^a^	6.91 ± 0.54^c^	26.20 ± 0.40^c^
MG	2.68 ± 0.84^b^	2.77 ± 1.21^b^	15.05 ± 1.64^a^	72.43 ± 16.63^a^
HQD	3.79 ± 0.37^a^	4.67 ± 2.12^ab^	7.83 ± 1.43^bc^	37.71 ± 9.08^c^
HQD-DZ	3.04 ± 0.44^ab^	3.14 ± 0.88^b^	7.14 ± 0.89^c^	34.41 ± 7.05^c^
HQD-GC	2.55 ± 0.91^b^	2.13 ± 1.06^b^	8.12 ± 1.37^bc^	42.55 ± 3.00^bc^
HQD-SY	3.31 ± 0.90^ab^	3.28 ± 1.22^b^	7.90 ± 0.61^bc^	39.25 ± 2.38^bc^
HQD-HQ	2.81 ± 0.68^ab^	3.16 ± 1.23^b^	9.27 ± 0.64^b^	49.78 ± 4.12^b^
ENR	3.29 ± 0.83^ab^	5.36 ± 1.92^a^	8.02 ± 1.12^bc^	34.71 ± 5.44^c^

The different letter superscript in the same column indicates that the difference is significant (*p* < 0.05).

CG, Control group; MG, Model group; DZ, Jujube, *Jujubae Fructus*; HQ, Scutellaria baicalensis, *Scutellariae Radix*; GC, liquorice, *Licoric*e; SY, Herbaceous peony, *Paeoniae Radix Alba*; HQD, decoction consists of DZ, HQ, GC and SY; HQD-DZ, HQD absent of DZ; HQD-GC, HQD absent of GC; HQD-SY, HQD absent of SY; HQD-HQ, HQD absent of HQ; ENR, Enrofloxacin group. (The dosage of Chinese medicine was 500 mg/kg).

### Lysozyme in serum

**Figure 4:**
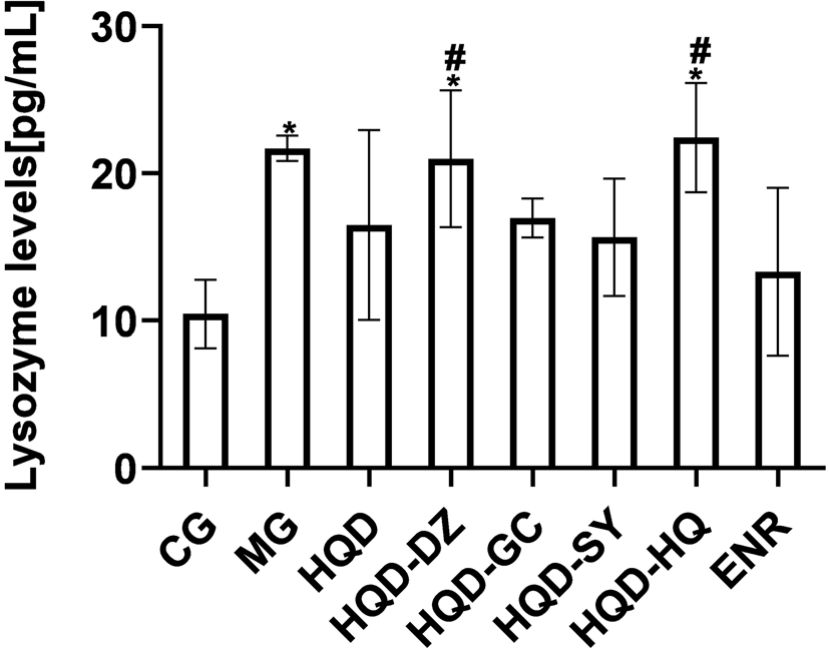
Lysozyme level of chicks was shown in the figure. Data are expressed as mean ± SEM. Statistical significance: *p < 0.05 versus model group, ^#^*p* < 0.05 versus HQD group. CG, Control group; MG, Model group; DZ, jujube, *Ziziphus jujuba*; HQ, Chinese skullcap, *Scutellaria baicalensis*; GC, licorice, *Glycyrrhiza glabra*; SY, white peony root, *Paeoniae radix alba*; HQD, decoction consists of DZ, HQ, GC and SY; HQD-DZ, HQD absent of DZ; HQD-GC, HQD absent of GC; HQD-SY, HQD absent of SY; HQD-HQ, HQD absent of HQ; ENR, Enrofloxacin group. (The dosage of Chinese medicine was 500 mg/kg).

Compared with CG, the level of LZM in the serum of chicks challenged with *E. coli* was increased, which was significantly reversed by HQD and ENR administration. HQD-GC and HQD-SY have the same effect as HQD, but HQD-DZ and HQD-HQ treatments could not reverse the increase in LZM protein level (Fig. 4).

### Inflammatory cytokines in serum

ELISA was conducted to determine the protein level of inflammatory cytokines in the serum. In MG, the protein levels of IL-1β, TNF-α and IL-10 in the serum were significantly higher (*p* < 0.05), when compared with CG. Compared with MG, the protein levels of IL-1β, TNF-α and IL-10 in the serum were significantly increased (*p* < 0.05) in HQD treatment, however, there was no significant difference compared with CG. HQD and ENR treatments had the same effect. The protein level of IL-6 was increased by *E. coli* challenge in the serum of chicks, when compared with CG. However, HQD treatment significantly reduced (*p* < 0.05) the protein level lower than the level of the control group. Compared with HQD group, the protein level of IL-6 was significantly increased (*p* < 0.05) in HQD-HQ, HQD-SY, HQD-DZ and HQD-GC treatments, while that of IL-1β and IL-10 did not show any significant difference. The level of TNF-α protein was significantly increased (*p* < 0.05) in HQD-SY and HQD-HQ treatments, when compared with HQD group. Moreover, the level of TNF-α protein in HQD-SY and HQD-HQ treatments were significantly higher (Table 2).

**Table 2:** The level of inflammatory factor in serum of chicks were shown in the table.

	IL-1β	IL-6	TNF-α	IL-10
CG	48.65 ± 8.61^c^	30.82 ± 2.91^c^	26.77 ± 2.10^d^	33.93 ± 4.82^bc^
MG	106.71 ± 5.38^a^	70.72 ± 2.39^a^	72.82 ± 3.58^a^	66.96 ± 2.39^a^
HQD	60.77 ± 14.61^bc^	20.11 ± 1.60^e^	24.38 ± 5.93^d^	37.95 ± 7.91^bc^
HQD-DZ	47.67 ± 7.22^c^	38.29 ± 2.40^b^	30.39 ± 3.17^cd^	24.68 ± 7.17^c^
HQD-GC	52.73 ± 25.10^c^	36.77 ± 3.73^b^	35.62 ± 7.79^cd^	30.90 ± 13.98^c^
HQD-SY	70.92 ± 29.76^b^	24.58 ± 2.86^d^	49.89 ± 11.95^b^	43.69 ± 28.43^b^
HQD-HQ	43.26 ± 10.91^c^	38.78 ± 3.04^b^	38.40 ± 2.57^c^	20.61 ± 8.13^c^
ENR	61.75 ± 20.39^bc^	28.33 ± 2.61^c^	27.21 ± 12.38^d^	31.99 ± 5.98^bc^

The different letter superscript in the same column indicates that the difference is significant (*p* < 0.05).

CG, Control group; MG, Model group; DZ, Jujube, *Jujubae Fructus*; HQ, Scutellaria baicalensis, *Scutellariae Radix*; GC, liquorice, *Licoric*e; SY, Herbaceous peony, *Paeoniae Radix Alba*; HQD, decoction consists of DZ, HQ, GC and SY; HQD-DZ, HQD absent of DZ; HQD-GC, HQD absent of GC; HQD-SY, HQD absent of SY; HQD-HQ, HQD absent of HQ; ENR, Enrofloxacin group. (The dosage of Chinese medicine was 500 mg/kg).

### Toll-like receptors in spleen and bursa of Fabricius

In order to further explore the effect of HQD on the immune function of chicks challenged with *E. coli*, the mRNA levels of TLR4, -5 and -15 in spleen and BF were detected. In MG, the mRNA levels of TLR4, -5 and -15 significantly higher (*p* < 0.05) in spleen, however, there was no significant difference in BF, when compared with CG. HQD and herb-absent treatments of the mRNA levels of TLR4, -5 and -15 significantly higher (*p* < 0.05) in BF, however, there was no significant difference in spleen, when compared with CG. Compared with HQD, ENR treatment of the mRNA levels of TLR4, -5 and -15 were no significant difference in spleen and BF, and there was no significant difference between herb-absent and HQD treatments (Table 3).

**Table 3:** The mRNA level of Toll-like receptor (TLR) in spleen and bursa of Fabricius of chicks were shown in the table.

	TLR4		TLR5		TLR15	
	Spleen	Bursa of Fabricius	Spleen	Bursa of Fabricius	Spleen	Bursa of Fabricius
CG	1.00 ± 0.05^b^	1.03 ± 0.26^b^	1.02 ± 0.20^b^	1.02 ± 0.22^b^	1.00 ± 0.13^b^	1.02 ± 0.23^b^
MG	5.46 ± 1.55^a^	0.98 ± 0.39^b^	10.36 ± 2.02^a^	0.67 ± 0.31^b^	5.88 ± 2.21^a^	1.11 ± 0.60^b^
HQD	1.51 ± 0.53^b^	5.75 ± 2.67^a^	1.66 ± 0.41^b^	3.29 ± 1.71^a^	1.51 ± 0.54^b^	6.54 ± 3.92^a^
HQD-DZ	1.22 ± 0.44^b^	7.11 ± 3.93^a^	1.20 ± 0.52^b^	3.13 ± 1.52^a^	1.19 ± 0.42^b^	6.42 ± 3.26^a^
HQD-GC	1.37 ± 0.40^b^	6.96 ± 4.08^a^	1.41 ± 0.54^b^	3.07 ± 2.64^a^	1.40 ± 0.53^b^	6.50 ± 3.95^a^
HQD-SY	1.66 ± 0.74^b^	6.00 ± 3.16^a^	1.73 ± 0.68^b^	3.33 ± 2.51^a^	1.74 ± 0.68^b^	6.70 ± 4.15^a^
HQD-HQ	1.18 ± 0.46^b^	7.19 ± 4.41^a^	1.18 ± 0.49^b^	3.10 ± 1.15^a^	1.07 ± 0.45^b^	6.47 ± 4.07^a^
ENR	1.51 ± 0.46^b^	6.14 ± 3.92^a^	1.60 ± 0.43^b^	3.25 ± 1.83^a^	1.51 ± 0.49^b^	6.62 ± 3.90^a^

The different letter superscript in the same column indicates that the difference is significant (*p* < 0.05).

CG, Control group; MG, Model group; DZ, Jujube, *Jujubae Fructus*; HQ, Scutellaria baicalensis, *Scutellariae Radix*; GC, liquorice, *Licoric*e; SY, Herbaceous peony, *Paeoniae Radix Alba*; HQD, decoction consists of DZ, HQ, GC and SY; HQD-DZ, HQD absent of DZ; HQD-GC, HQD absent of GC; HQD-SY, HQD absent of SY; HQD-HQ, HQD absent of HQ; ENR, Enrofloxacin group. (The dosage of Chinese medicine was 500 mg/kg).

### Gut microbiota in chick feces

#### Structural modulation of gut microbiota

Compared with CG, the alpha diversity was significantly reduced (*p* < 0.05) in MG, which was reversed by HQD treatment. There was no significant difference in CG and HQD treatments. The Chao1 index were significantly reduced (*p* < 0.05) in HQD-HQ, HQD-DZ and HQD-SY treatments, when compared with HQD group. Moreover, the Shannon index of HQD-DZ treatment was significantly decreased (*p* < 0.05) compared with HQD group. The Chao1 index and the Shannon index were significantly reduced (*p* < 0.05) in ENR treatment, when compared with CG (Table 4).

**Table 4:** The diversity of microbial in group of chicks were shown in the table.

	Chao1	Shannon
CG	995.70 ± 133.14^a^	8.79 ± 0.26^a^
MG	638.46 ± 43.52^b^	6.87 ± 0.13^b^
HQD	884.87 ± 119.90^a^	8.55 ± 1.26^ab^
HQD-DZ	518.60 ± 42.76^b^	2.78 ± 0.77^c^
HQD-GC	823.83 ± 268.85^ab^	8.53 ± 0.78^ab^
HQD-SY	455.77 ± 127.88^b^	6.76 ± 1.06^b^
HQD-HQ	565.00 ± 86.09^b^	7.38 ± 2.01^ab^
ENR	338.13 ± 35.36^b^	5.03 ± 0.76^b^

The different letter superscript in the same column indicates that the difference is significant (*p* < 0.05).

CG, Control group; MG, Model group; DZ, Jujube, *Jujubae Fructus*; HQ, Scutellaria baicalensis, *Scutellariae Radix*; GC, liquorice, *Licoric*e; SY, Herbaceous peony, *Paeoniae Radix Alba*; HQD, decoction consists of DZ, HQ, GC and SY; HQD-DZ, HQD absent of DZ; HQD-GC, HQD absent of GC; HQD-SY, HQD absent of SY; HQD-HQ, HQD absent of HQ; ENR, Enrofloxacin group. (The dosage of Chinese medicine was 500 mg/kg).

**Figure 5:**
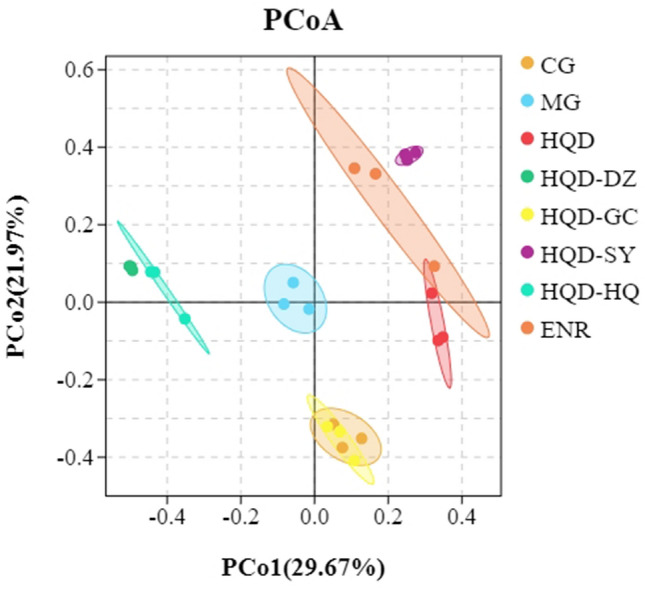
Analysis of intestinal microorganism of chicks were shown in the figure. CG, Control group; MG, Model group; DZ, jujube, *Ziziphus jujuba*; HQ, Chinese skullcap, *Scutellaria baicalensis*; GC, licorice, *Glycyrrhiza glabra*; SY, white peony root, *Paeoniae radix alba*; HQD, decoction consists of DZ, HQ, GC and SY; HQD-DZ, HQD absent of DZ; HQD-GC, HQD absent of GC; HQD-SY, HQD absent of SY; HQD-HQ, HQD absent of HQ; ENR, Enrofloxacin group. (The dosage of Chinese medicine was 500 mg/kg).

A plateaued rarefaction curve of OTUs indicated that the sequencing depth covered all the species in the samples. *E. coli* inoculation increased the OTUs, when compared with CG, which were reversed by HQD and ENR administration. Principal coordinate analysis (PCoA) showed similarity among samples, with similarity indicated by distance in the diagrams. *E. coli* inoculation altered the composition and structure of the gut microbiota according to PCoA. Treatment with HQD partially inhibited *E. coli*-induced changes in the gut microbiota (Fig. 5).

#### Structure difference analysis of the gut microbiota

**Figure 6:**
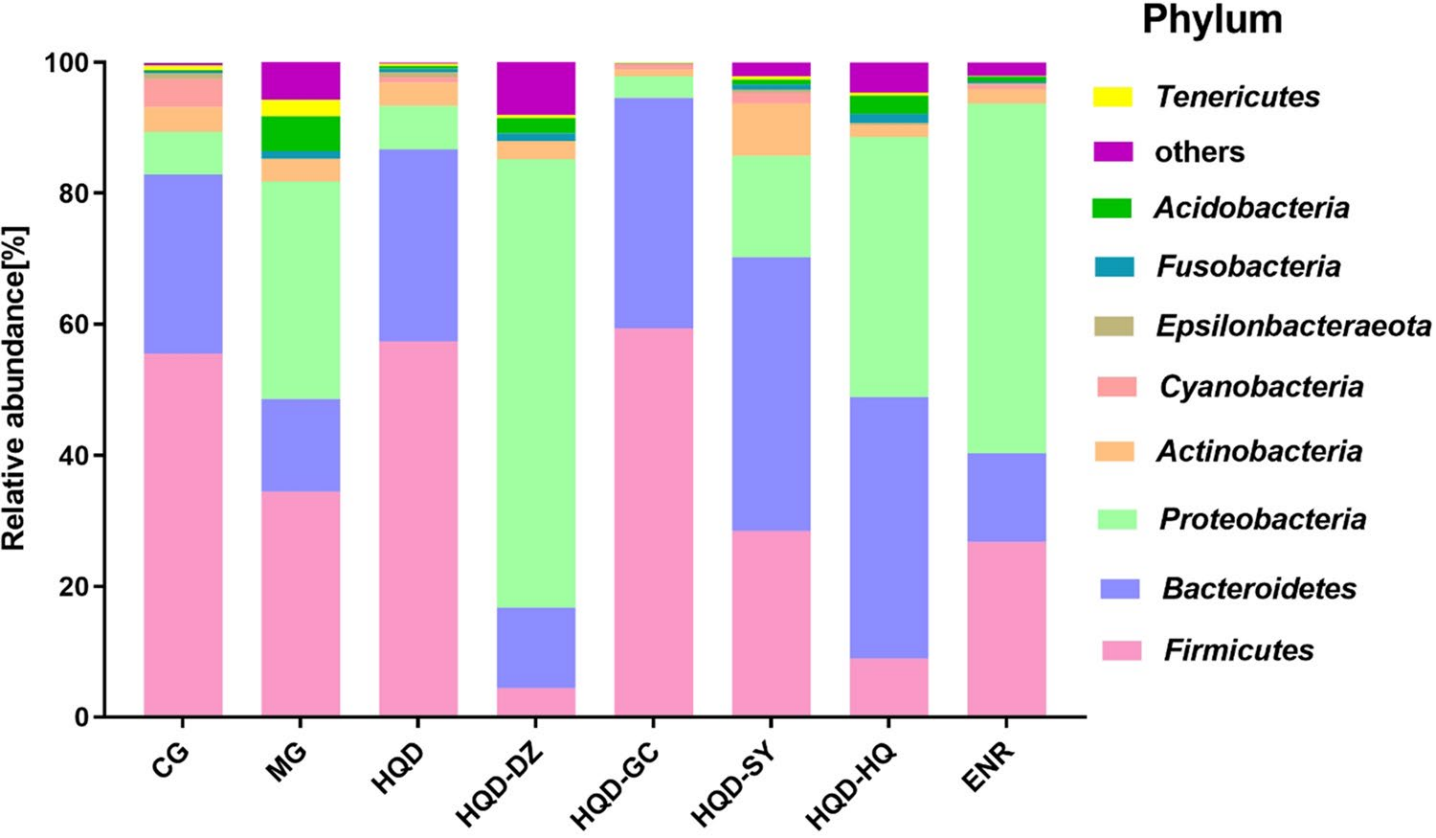
Phylum level analysis of intestinal microorganism relative abundance of chicks were shown in the figure. CG, Control group; MG, Model group; DZ, jujube, *Ziziphus jujuba*; HQ, Chinese skullcap, *Scutellaria baicalensis*; GC, licorice, *Glycyrrhiza glabra*; SY, white peony root, *Paeoniae radix alba*; HQD, decoction consists of DZ, HQ, GC and SY; HQD-DZ, HQD absent of DZ; HQD-GC, HQD absent of GC; HQD-SY, HQD absent of SY; HQD-HQ, HQD absent of HQ; ENR, Enrofloxacin group. (each color represents one bacterial phylum). (The dosage of Chinese medicine was 500 mg/kg).

The gut microbiota community structure was reported using histograms at the phylum and genus level. All samples contained abundant *Firmicutes*, *Bacteroidetes* and *Proteobacteria*. In MG, the relative abundance of *Bacteroidetes* and *Firmicutes* were significantly decreased (*p* < 0.05) and increased (*p* < 0.05) the proportion of *Proteobacteria*, when compared with CG. These alterations were significantly reversed by HQD administration. Compared with HQD group, the HQD-DZ significantly increased (*p* < 0.05) in the abundance of *Proteobacteria*. The ENR treatment could not recover the changes in the abundance of dominant bacteria compared with MG. In contrast, the abundance of *Proteobacteria* was further increased (Fig. 6).

**Figure 7:**
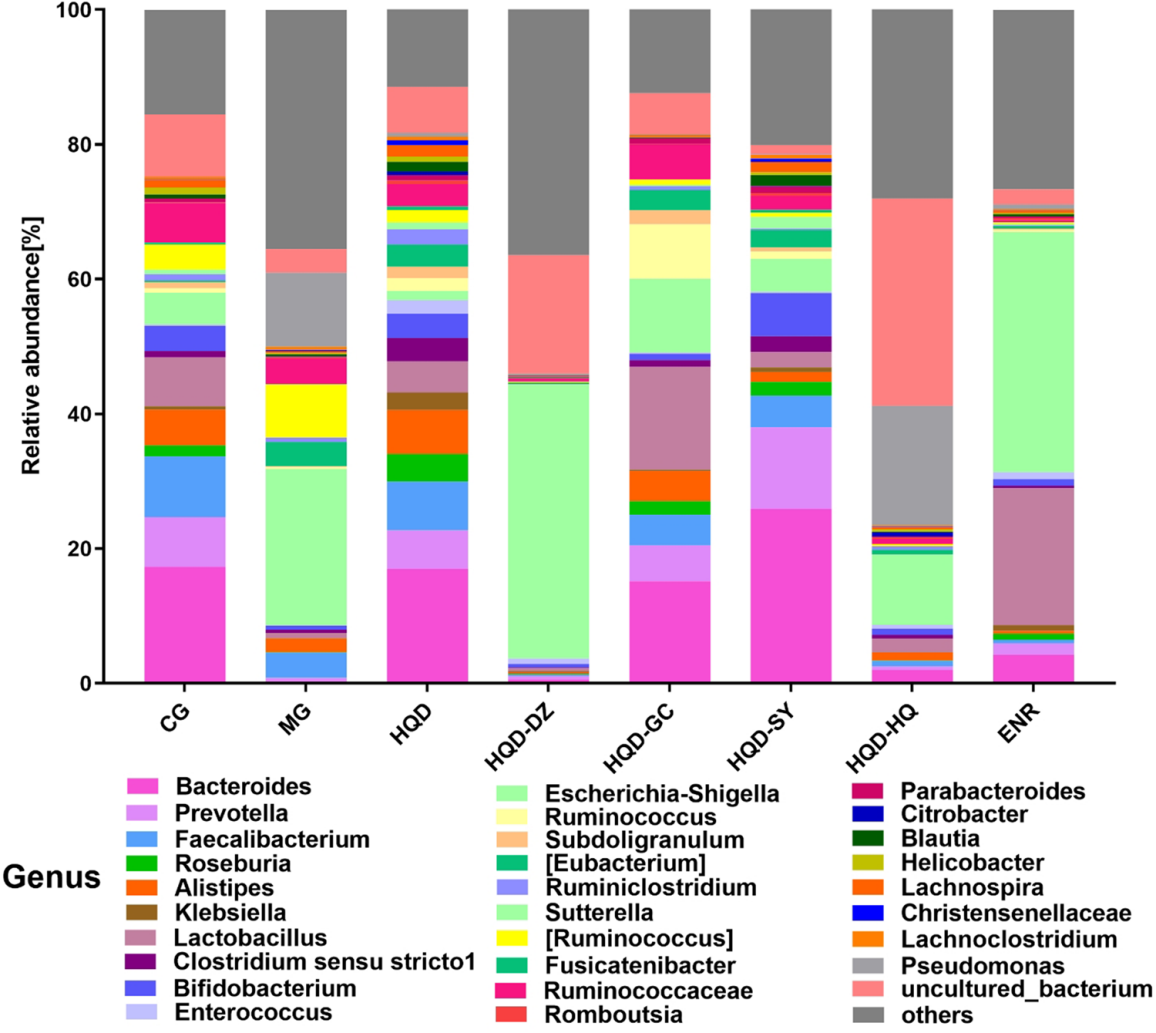
Genus level analysis of intestinal microorganism relative abundance of chicks were shown in the figure. CG, Control group; MG, Model group; DZ, jujube, *Ziziphus jujuba*; HQ, Chinese skullcap, *Scutellaria baicalensis*; GC, licorice, *Glycyrrhiza glabra*; SY, white peony root, *Paeoniae radix alba*; HQD, decoction consists of DZ, HQ, GC and SY; HQD-DZ, HQD absent of DZ; HQD-GC, HQD absent of GC; HQD-SY, HQD absent of SY; HQD-HQ, HQD absent of HQ; ENR, Enrofloxacin group. (each color represents one bacterial genus). (The dosage of Chinese medicine was 500 mg/kg).

More than 30 genera were identified in all samples. *Bacteroides*, *Faecalibacterium, Lactobacillus* and *Prevotella* were dominant communities in the control group but were reduced by *E. coli* inoculation. Compared with CG, *Escherichia-Shigella* becomes the dominant genus, but were reduced by HQD treatment. The gut microbiota community structure of HQD, HQD-GC and HQD-SY were like that of CG. Compared with HQD group, the proportion of *Escherichia-Shigella* were significantly increased in HQD-DZ and HQD-HQ treatments (Fig. 7)*.*

## Discussion

Considering the incidence of multi-drug resistant APEC and its potential risk for both human and animal health, it is imperative to develop alternative strategies to reduce *E. coli* -infection-induced deleterious effect^11,12^. Previous studies have shown that HQD has no inhibitory effect on *E. coli* in vitro, but this effect has been found in vivo studies^13^. Therefore, it is speculated that the positive effect of HQD on *E. coli* inoculation in chicks is dependent on the intestinal metabolism, regulation of intestinal microflora structure and its immune function.

In previous study, the method of intramuscular injection was used to inoculate the chicks, which allowed *E. coli* to cross the first immune barrier and enter the bloodstream, and then initiate the secretion of inflammatory factors by epithelial cells and macrophages^14^. The cytokine genes IL-8, IL-1β, IL-6, TNF-α and IL-10 are the regulators of pro- and anti-infammatory immune responses. NF-κB plays an important role as a transcription factor in inflammatory and immune responses in many inflammatory diseases, and is involved in the transcription of a variety of proteins, including mammalian RelA (P65), RelB, P50 and P52^15,16^. In the present study, *E. coli* inoculation led to severe perihepatitis and pericarditis, and increasing mortality rates, decreasing ADG, and raising organ indexes of heart and liver in the inoculated chicks, which was attenuated by HQD administration. Moreover, the experimental infection resulted in increased LZM, as well as enhanced levels of inflammatory factors, including IL-1β, IL-6, TNF-α and IL-10 in serum, indicating activation of inflammatory response following *E. coli* inoculation. While, HQD decreased the serum LZM, IL-1ß, TNF-α, IL-10, IL-6 level so to consequently improve the immune function. However, there was no significant difference in HQD-SY, HQD-DZ and HQD-HQ treatments compared with HQD, indicating that SY, DZ and HQ play a key role in reducing the level of inflammatory factors.

It is worth noting that *E. coli* inoculation led to increased level of IL-10, which has an anti-inflammatory and immunosuppressive effect. Some researchers believe that anti-inflammatory cytokine IL-10 can reduce the intensity of inflammatory response in severe infection. On the other hand, the inhibition of the body's defense ability leads to the persistence of microbial infection^17^. Therefore, it is believed that the increase of IL-10 in an infection may be a manifestation of severe injury of the body^18^. In present study, *E. coli* inoculation increased protein level of IL-10, which may indicate that *E. coli* causes a severe damage of the body.

TLRs expressed by mammalian and avian immune system cells have the capability to recognize pathogen-associated molecular patterns. Activation of TLRs signaling pathway plays an important role in defensing against invading pathogens. TLRs could identify the microbe related molecular patterns, activate MyD88-dependent signaling pathway, activate transcription factor NF-kB producing IL-1ß, IL-6, TNF-α and other cytokines, and enhance the immune function of the body^19–21^. In chicks, TLR1A, -1B, -2A, -2B, -3, -4, -5, -7, -15 and -21 are expressed by cells of the immune system as well as by epithelial cells. Recognition of ligands by TLRs enhances the production of cytokines and increases expression of costimulatory molecules that modulate adaptive immune responses to exhibit both pro- and anti-inflammatory properties^22,23^, for example TLR4 protein exhibit immune response at tissue level to fight against the invaded pathogens in chicks. Among that, the spleen is the biggest peripheral immune organ, and BF and thymus are central lymphoid organs in the immune system of poultry.

In the present study, *E. coli* inoculation led to enhanced mRNA levels of TLR4, -5 and -15 in spleen, indicating activation of the innate immune system in the spleen following the infection. These up-regulated TLRs in the spleen were accompanied by an increase of the serum inflammatory factors including IL-1β, IL-6, TNF-α and IL-10. The expression of TLRs in spleen of chicks treated with HQD was down-regulated accompanying an increase of all the serum inflammatory factors so as to improve the immune function of infected chicks. With regard to the mRNA levels of TLRs in BF, though there was no significant difference between CG and MG. The expression of TLRs in BF of chicks treated with HQD were up-regulated, indicating that HQD regulates the anti-infection ability by increasing the mRNA levels of TLR4, -5 and -15, further decreasing the serum LZM and IL-1β, TNF-α, IL-10, IL-6 level of chicks suffered *E. coli* infection. In brief, it is suggested that HQD could down-regulate the expressions of TLR4, -5 and -15 in spleen but up-regulate that of TLRs in BF, so together to reduce the levels of inflammatory factors. While, there was no difference between the expression of TLRs of HQD and all treatments of herb absent both in spleen and BF, as indicating that HQD plays a role in regulating TLRs as a whole and then the inflammatory factors.

Structural changes in the gut microbiota have been reported in *E. coli* models^24^. Intestinal microflora plays an important role in the general health of the host^22,25^, which is closely related to the host's immunity, metabolism, digestion, absorption and susceptibility to diseases^26^. Previous studies have reported that *Firmicutes* can metabolize and produce butyric acid in the intestinal tract, which provides energy for the growth and development of the intestinal cells^27^. Also, it influences the fermentation of carbohydrates and polysaccharides to improve the transformation of nutrients and energy in the animal body^28^. ENR has a good therapeutic effect, but it leads to dysregulation of intestinal flora, which is related to its extensive bactericidal action. It leads to the destruction of a large number of bacteria in the gut, which releases more LPS, triggering an inflammatory response, which also explains the increased level of inflammatory cytokines^29^. Therefore, it is of great significance to maintain the structural stability and diversity of intestinal flora. In the present study, the abundance and diversity of intestinal microflora in chicks inoculated with *E. coli* were significantly decreased (*p* < 0.05). It is a marker of intestinal microbial dysregulation. HQD treatment had therapeutic effect on the decrease of diversity of bacteria caused by *E. coli* inoculation. HQD-HQ, HQD-DZ, HQD-GC and HQD-SY treatments had varying degrees of influence on the three phylum levels. These results indicate that each herb in HQD influences structural segregation of the gut microbiota, in which DZ plays an important role in maintaining the intestinal microflora structure of chicks challenged with *E. coli*. The results showed that *E. coli* broke the immune barrier, colonized and proliferate in mucous membrane, which produced toxin to cause damage to different organs following the blood circulation. *E. coli* challenged the immune function of the body, and increased the prevalence of a large number of opportunistic pathogens in the intestine. Finally, inoculation with *E. coli* decreased the diversity of intestinal microflora and disrupted its balance. Fortunately, HQD can effectively attenuate this phenomenon. When compared with HQD group, the Shannon index in HQD-DZ treatment was significantly decreased, indicating that DZ had an important role in maintaining the intestinal microflora structure of chicks challenged with *E. coli*.

The analysis of the intestinal microflora structure on the phylum level in each experimental treatment showed significant changes including an increase in a proportion of *Proteobacteria*, and a decrease in a proportion of *Firmicutes*. *Proteobacteria* is considered to be a marker of intestinal microbiota imbalance, and a large number of *Proteobacteria* in the gut reflects stunted growth or an unstable intestinal microbiota structure^30^. Administration of HQD attenuates this change and keeps the intestinal microflora in balance. The ENR treatment could not recover the changes in the abundance of dominant bacteria compared with MG. In contrast, the abundance of *Proteobacteria* was further increased. The results suggest that HQD treatment is more beneficial than ENR in the treatment of *E. coli* infection. HQD-DZ treatment increased the prevalence of harmful bacteria, namely *Proteobacteria*, which indicates that DZ in HQD play a key role in keeping fecal microbiome balance in the phylum level.

The genus structure of the microflora were changed by *E. coli* inoculation. The proportion of *Proteobacteria, Faecalibacterium, Lactobacillus* and *Prevotella* were decreased, and *Escherichia-Shigella* became the dominant genus, as was reversed by HQD treatment similar to control group, indicating that the proportions of most bacteria returned to control level following HQD treatment. The proportions changed after HQD, HQD-GC and HQD-SY treatment, these became similar to the microbiome structure in CG, indicating that HQD, HQD-GC and HQD-SY had protective effect on the structural changes caused by *E. coli* inoculation. Moreover, HQD-DZ and HQD-HQ increased the prevalence of *Escherichia-Shigella*, indicating that DZ and HQ in HQD had a certain regulating effect on the increase of *Escherichia-Shigella* and play a key role in keeping fecal microbiome balance in the genus level.

## Conclusions

In conclusion, HQD application improve the immune function by down-regulating the mRNA expression of TLR4, -5 and -15 in the spleen, further decreasing the serum LZM and IL-1β, TNF-α, IL-10, IL-6 level, and reverse the change of the fecal microbiome in chicks challenged with *E. coli*. Moreover, SY and DZ play a key role in reducing the level of inflammatory factors and keep the fecal microbiome balance respectively. More importantly, HQ is indispensable in HQD, not only play a key role in reducing the level of inflammatory factors, but also in keeping the balance of fecal microflora.

## Materials and methods

### Chicks and treatments

A total of 130 1-day-old male Isa brown chicks were randomly assigned into one of 13 treatment groups with 10 chicks per group. Chicks in CG were fed a basal diet to meet nutritional requirements of chicks during the whole experimental period; chicks in MG group were inoculated with *E. coli* without feed supplementation. *E. coli* inoculated chicks in ENR, HQD, HQD-HQ, HQD-DZ, HQD-GC and HQD-SY were supplemented with ENR 10 mg/kg·BW, HQD granules 250 and 500 mg/kg·BW, HQD-HQ granules 250 and 500 mg/kg·BW, HQD-DZ granules 250 and 500 mg/kg·BW, HQD-GC granules 250 and 500 mg/kg·BW and HQD-SY granules 250 and 500 mg/kg·BW respectively. Chicks were transferred to housing chambers with pine shavings on a concrete floor in a temperature-controlled environment (Temperature 35 ℃, humidity 60–70%) and housed in two separate chambers with identical environmental conditions to separate the control group from infected chicks. All supplement drugs were administered after intramuscular injection of *E. coli* at 27 days of age, and they were added up to 32 days of age. All the chicks had free access to feed and drinking water during the whole experimental period. Behaviour, state of feathers and clinical symptoms of chicks were observed and recorded every day. Moreover, the records of body weight, feed intake and daily mortality were taken as well. The experimental animal protocol for this study was approved by the Animal Care and Use Committee of China Agricultural University.

### *E. coli* O78 rejuvenation and experimental infection

To rejuvenate, the *E. coli* O78 strain (repository number: CVCC1490; China Veterinary Microorganism Strains Preservation Management Center, Beijing, China), was used to inoculate in the chicks which were sacrificed after and their livers were collected. By streaking the liver swab, *E. coli* O78 was isolated on MacConkey agar plate at 37 °C for 24 h and frozen at − 80 °C^31^. The bacterial inocula were prepared by 24 h of growth on Luria–Bertani (LB) broth (Difco, Sparks, USA) at 37 °C. The bacteria were harvested and washed twice, and the counts were adjusted to 0.6 × 10^9^ CFU/mL to get sufficient bacteria for intramuscular injection to chicks with 0.5 mL of inoculum at 27 days of age^32^.

### Preparation for granules of HQD and herb-absent HQD

According to the prescription of HQD, HQ, SY, DZ and GC were soaked in a ratio of 3:2:2:2 for 60 min and boiled for 40 min. After filtration, the residue of the HQ was dried in a drying oven, then the grinded residue was mixed with the concentrated filtrate in the pelletizer, and then cut to wet granules following desiccation. In the granule preparation of HQD-SY, HQD-DZ, HQD-GC and HQD-HQ, SY, DZ, GC and HQ were absent in decoction, respectively.

### Visceral index

At 32 days of age, the live weight of chicks was measured and after that they were sacrificed, and liver, heart, spleen, and BF were collected to calculate the chicks’ visceral index. The formula to calculate the visceral index is Visceral index (g/kg) = visceral weight (g)/live weight (kg).

### Enzyme-linked immunosorbent assay (ELISA)

At 32 days of age, to evaluate the effects of drugs on innate immune substances and cytokines in chicks with colibacillosis, the blood was collected, and the serum was separated. The level of LZM, TNF-α, IL-1β, IL-6, and IL-10 in serum were quantified using ELISA kits (Wuhan Beinley Biotechnology Co., LTD, Shanghai, China) following manufacturer's instructions. Three samples from each group were analyzed in triplicate. The results were expressed as content of LZM or cytokines per mL of serum in chicks.

### Quantitative real-time PCR (qPCR)

At 32 days of age, to evaluate the effect of drugs on TLRs expression in immune organs of chicks with colibacillosis, the BF and spleen was collected. Total RNA was extracted from the spleen and BF using Trizol reagent (TaKaRa Co., Tokyo, Japan) following manufacturer's instructions. According to the Bio-Rad kit (Wuhan ServiceBio Technology Co., Ltd., China), 1 μL of RNA was taken for qPCR reaction. Gapdh gene was used as a housekeeping gene to normalize the CT values. The full-length sequence of Gapdh gene mRNA on NCBI was used to design specific primers, which were synthesized by Wuhan ServiceBio Technology Co., Ltd. Three samples from each group were analyzed in triplicate. Primer sequences (Wuhan ServiceBio Technology Co., Ltd., China) used are shown in the Table 5.

**Table 5:** Primer sequences.

Primer	Primer sequences (5′–3′)
Gapdh-F	CTGGGGCTCATCTGAAGGGT
Gapdh-R	GGACGCTGGGATGATGTTCT
TLR4-F	CCAACCCAACCACAGTAGCAT
TLR4-R	ATCACCCATTCTTGGTCTTTGC
TLR5-F	TCTGACATACGATGACTGCGAT
TLR5-R	TGACATCAGAAACATCAGAAGGGT
TLR15-F	CGACAACGCCATCACTACCATA
TLR15-R	GTTAGCACCAGAACGACAAGGA

### Fecal microflora analysis

#### Extraction of fecal DNA

At 32 days of age, the intestinal flora of all groups was studied. Feces of each group of animals was collected and placed into a sterile cryopreservation tube, which was immediately frozen with liquid nitrogen and stored at − 80 °C for use. Total genomic DNA from Stool was extracted and purified using DNeasy PowerSoil kit (100) (Qiagen, Hilden, Germany). The integrity and quantity of DNA were determined by agarose gel electrophoresis and spectrophotometry. The amount of 500 ng of the purified DNA was extracted from each sample for subsequent polymerase chain reaction (PCR) and 16S rDNA sequencing.

#### Sequencing of 16S rDNA

Samples were taken and diluted to 10 ng/μL with sterile water. Using the diluted genomic DNA as a template, V3 + V4 hypervariable sequences of 16S rDNA were amplified by PCR with Takara Ex Taq (TaKaRa Co., Tokyo, Japan) with barcode-modified universal primers (forward: 343F, 5′-TACGGRAGGCAGCAG-3′; reverse: 798R, 5′-AGGGTATCTAATCC T-3′). PCR mixtures contained 15 μL of 2 × Gflex PCR Buffer, 2 μL of primer mix (5 pmol/μL), 5 μL of diluted DNA template (10 ng/μL), 0.6 μL of Tks Gflex DNA Polymerase (1.25 U/μL) in a total reaction volume of 30 μL. PCR conditions were as follows: initial denaturation at 94 °C for 5 min, 26 cycles of denaturation at 94 °C for 30 s, annealing at 56 °C for 30 s, and elongation at 72 °C for 20 s, with a final elongation step at 72 °C for 5 min. Amplified products were separated on 2% agarose gels, the purified product was used as a second PCR template for amplification. The above steps were repeated for purification, and then Qubit dsDNA Assay Kit (Life Technologies, CA, USA) was used for quantitative detection. According to the concentration of PCR products, equal amounts of samples were mixed, and the barcoded V3 + V4 amplicons were sequenced by the Illumina MiSeq (Illumina, USA) (Shanghai OE Biotech. Co., Ltd., China) (“Supplementary information”).

The species diversity in different groups was evaluated by taxonomic analysis of OTU, moreover, the composition of flora at phylum and genus level were analyzed to explain the differences of flora structure among different groups. In this study, the microorganism α-community abundance index (Chao1) and community diversity index (Shannon) were used for diversity analysis. The larger the Chao1 index, the higher the flora richness in the sample. The larger the Shannon index, the higher the intestinal flora diversity^33^.

#### Bioinformatics analysis

Raw sequences were denoised using Trimmomatic v.0.3.6 and FLASH v.6.0. software and filtered according to their barcodes and primer sequences with QIIME v.1.5.0. Chimeras were identified and excluded using the UCHIME algorithm v.4.2.40. Optimized, high-quality sequences were clustered into OTUs at 97% sequence identity against a subset of the Silva 16S sequence database (Release 119 1). Taxon-dependent analysis was carried out using the Ribosomal Database Project (RDP) naive Bayesian classifier, with an 80% bootstrap cutoff. Alpha diversity (Shannon and Simpson indices), abundance (Chao1 and ACE indices), and Goods coverage and rarefaction were analyzed with mothur v.1.31.2. Principle coordinates analysis (PCoA) was conducted to visualize differences in nasal mucosa community composition. PCoA plots were generated based on Bray–Curtis indices. The linear discriminant analysis effect size (LEfSe) algorithm was used to identify the taxa responsible for the differences between the treatment and control groups. The biomarkers used in the present study had an effect-size threshold of two.

### Statistical analysis

Statistical analysis of data from ELISA and qPCR assays was carried out using a one-way analysis of variance (ANOVA) to detect differences between mean values, which were then further analyzed for significance with LSD test. The chi-square test was used to analyze the differences in mortality. In all cases, p < 0.05 was considered statistically significant.

All the methods were performed in accordance with relevant guidelines and regulations.

### Ethics approval and consent to participate

The experimental animal protocol for this study was approved by the Animal Care and Use Committee of Shanxi Agricultural University.

### Guidelines and regulations

This study is reported in accordance with ARRIVE guidelines.

## Supplementary Information

Supplementary Information.

## Abbreviations

DZ: Jujube, *Ziziphus jujuba*
HQ: Chinese skullcap, *Scutellaria baicalensis*
GC: Licorice, *Glycyrrhiza glabra*
SY: White peony root, *Paeoniae radix alba*
HQD: Chinese medicine of decoction which consists of DZ, HQ, GC and SY
HQD-DZ: HQD absent of DZ
HQD-HQ: HQD absent of HQ
HQD-GC: HQD absent of GC
HQD-SY: HQD absent of SY
