# Author Correction: Influence of Huangqin Decoction on the immune function and fecal microbiome of chicks after experimental infection with *Escherichia coli* O78

**DOI:** 10.1038/s41598-022-24320-4

**Published:** 2022-11-18

**Authors:** Junyan Wang, Rui Li, Minai Zhang, Chensheng Gu, Haili Wang, Jianjian Feng, Linjie Bao, Yihe Wu, Shuming Chen, Xichun Zhang

**Affiliations:** 1 College of Veterinary Medicine, Shanxi Agricultural University, Taigu, People’s Republic of China; 2 College of Pharmaceutical and Food Engineering, Shanxi University of Chinese Medicine, Yuci, People’s Republic of China; 3 Technical Center of Taiyuan Customs, Taiyuan, Shanxi People’s Republic of China

Correction to: *Scientific Reports* 10.1038/s41598-022-20709-3, published online 05 October 2022

The original version of this Article contained an error in the order of the author names, which was incorrectly given as Junyan Wang, Rui Li, Minai Zhang, Chensheng Gu, Haili Wang, Jianjian Feng, Linjie Bao, Yihe Wu, Xichun Zhang & Shuming Chen.

The original Article has been corrected.

